# Prognostic models to predict overall and cause-specific survival for patients with middle ear cancer: a population-based analysis

**DOI:** 10.1186/1471-2407-14-554

**Published:** 2014-08-01

**Authors:** Weidong Shen, Naoko Sakamoto, Limin Yang

**Affiliations:** Department of Otolaryngology, The Institute of Otolaryngology, Head and Neck Surgery, Chinese PLA General Hospital, Beijing, P.R. China; Department of Public Health, Juntendo University, Tokyo, Japan; National Center for Child Health and Development, Epidemiology and Clinical Research Center for Children’s Cancer, Tokyo, Japan; Division of Allergy, Department of Medical Subspecialties, Medical Support Center for Japan Environment and Children’s Study (JECS), National Center for Child Health and Development, 2-10-1 Okura, Setagaya-ku, Tokyo, 157-8535 Japan

**Keywords:** Middle ear cancer, Nomogram, Overall survival, Cause-specific survival

## Abstract

**Background:**

The purpose of this study was to evaluate the survival outcome for middle ear cancer and to construct prognostic models to provide patients and clinicians with more accurate estimates of individual survival probability.

**Methods:**

Patients diagnosed with middle ear cancer between 1983 and 2011 were selected for the study from the Surveillance Epidemiology and End Results Program. We used the Kaplan-Meier product limit method to describe overall survival and cause-specific survival. Cox proportional hazards models were fitted to model the relationships between patient characteristics and prognosis. Nomograms for predicting overall survival and cause-specific survival were built using the Cox models established.

**Results:**

The entire cohort comprised 247 patients with malignant middle ear cancer. Median duration of follow-up until censoring or death was 25 months (range, 1–319 months). Five-year overall survival and cause-specific survival were 47.4% (95% Confidence Interval (CI), 41.2% to 54.6%) and 58.0% (95% CI, 51.6% to 65.3%), respectively. In multivariable analysis, age, histological subtype, stage, surgery and radiotherapy were predictive of survival. The bootstrap corrected c-index for model predicting overall and cause-specific survival was 0.73 and 0.74, respectively. Calibration plots showed that the predicted survival reasonably approximated observed outcomes.

**Conclusion:**

The models represent an objective analysis of all currently available data. The resulting models demonstrated good accuracy in predicting overall survival and cause-specific survival. Nomograms should thus be considered as a useful tool for predicting clinical prognosis.

## Background

Malignant tumors of the temporal bone are rare and account for <0.2% of all tumors of the head and neck [1]. The most common tumors include squamous cell carcinoma (SCC), adenocarcinoma, basal cell carcinoma, adenoid cystic carcinoma, rhabdomyosarcoma, and Langerhans histocytosis X (LHX). Primary carcinoma of the middle ear represents a small subset of temporal bone carcinomas [2]. The incidence of middle ear cancer was approximately 0.18 per million people in the United States in 2011, according to the Surveillance Epidemiology and End Results (SEER) [3]. The diagnosis is often delayed because the disease may be masked by other ear symptoms. The complex anatomy of the temporal bones, delayed presentation and diagnosis make the surgical management difficult [1]. Although treatment has been improving over the decades, the prognosis remains poor [4].

Because of the rarity of malignant tumors of the middle ear, most reports published previously were based on case series from a single institution, and most of the literature does not separate carcinomas of the external auditory canal from primary carcinomas of the middle ear [2]. The prognostic results therefore lack uniformity. To make advances in the management of patients with middle ear cancer, we should improve our understanding of this disease process.

The SEER is a large population-based database that has been used to provide epidemiological and prognostic information about many types of cancers. Using a population-based database can avoid the limitations of a small sample size and the selection or treatment bias associated with analysis of a single institution’s clinical data. The purpose of this study was to evaluate the survival outcomes of middle ear cancer using the SEER database. We also developed simple nomograms based on the prognostic models established.

## Methods

**Figure 1:**
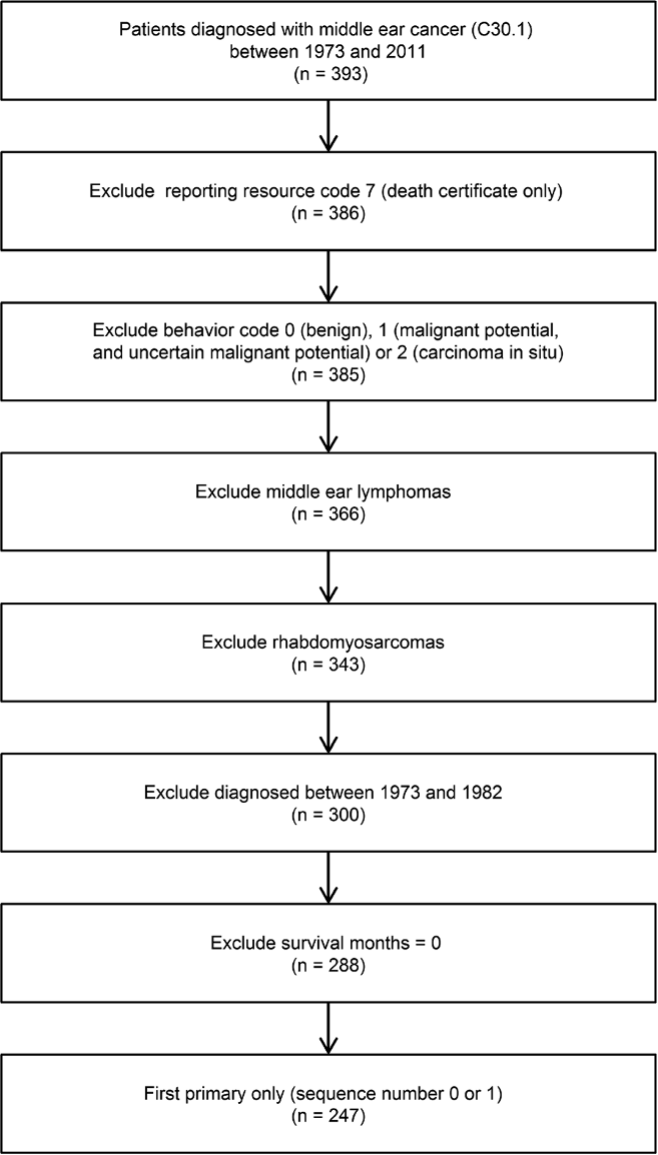
**Flow chart for creation of the Surveillance Epidemiology and End Results (SEER) data set.**

The SEER program of the National Cancer Institute is the largest population-based cancer registry in the United States. The SEER registries collect data on patient demographics, primary tumor site, stage, tumor morphology, and treatment for all cancer patients, covering approximately 28% of the US population. For this research, the recently released SEER (1973–2011) database was used for case extraction [5]. All patients diagnosed with middle ear cancer as their first malignancy were selected (site code C30.1). Patients were excluded from the study if the tumor was identified on the death certificate only. Middle ear lymphomas and rhabdomysarcomas were also excluded. Because there is no SEER stage information before 1983, we also excluded patients diagnosed between 1973 and 1982. Detailed data selection is shown in Figure 1.

Variables in the analysis included: age at diagnosis; sex; race; year of diagnosis; histological subtype; cancer stage; surgery and radiotherapy. The American Joint Committee on Cancer (AJCC) has not yet produced a staging system for ear and temporal bone cancer. Here, the clinical stage of cancer was grouped using the historical stage coded by SEER: ‘localized’ was defined as a tumor confined to the organ; ‘regional’ was defined as a neoplasm that had extended into surrounding organs or tissues, or into regional lymph nodes or by a combination of extension and regional lymph nodes, and ‘distant’ was defined as a tumor that had spread to parts of the body remote from the primary tumor. The histological subtype was re-grouped as squamous cell carcinoma, adenocarcinoma and others.

Missing values were imputed with the ‘tanscan’ function of the regression modeling strategies (rms) package [6]. Patients in the cohort were followed for vital status until the earliest of the following dates: death; last contact if before December 31, 2011 or December 31, 2011 if the date of last contact was after December 31, 2011.

Median follow-up was defined as the median observed survival time among all patients. We used the Kaplan-Meier product limit method to describe overall survival (OS) and cause-specific survival (CSS). The Cox proportional hazards model was fitted to model the relationships between patient characteristics and survival. Variables used for building the multivariate regression model were chosen based on known clinically prognostic factors and availability in the SEER registry. We tested the proportional hazard assumption for each variable in the model. The restricted cubic splines with three knots at the 10%, 50% and 90% empirical quantiles were fitted to model age variables. Interaction between histological type and surgery was evaluated in the model. To avoid overfitting, we used a backward model selection technique based on Akaike information criterion (AIC) to reduce variables in the model [6]. The Wald test was conducted to find which predictors were significant in the model. Nomograms were developed on plain paper based on the reduced models.

The prediction models were internally validated. Bootstrap validation was performed to evaluate the performance of the model. A concordance index (c-index) was calculated by bootstrapping with 200 resamples to obtain an unbiased estimate of the ability of the model to discriminate among patients. Subsequently, we compared the predicted probability of survival versus actual survival also using 200 bootstrap resamples.

All statistical analysis was performed using R version 3.0.0 software (Institute for Statistics and Mathematics, Vienna, Austria; http://www.r-project.org) [7]. The R package rms was used for modeling and developing the nomogram [8]. All *P* values presented in this article were calculated based on a two-sided statistical test.

## Results

### Overall and cause-specific survival

A total of 247 cases of middle ear cancer were eligible for inclusion, and 143 patients died during the study period. Of these, 97 of 143 deaths were classified as cause-specific death. Table 1 shows patients and tumor characteristics. Sixty-eight patients (27.5%) were younger than 50 years, 94 patients (38.1%) were 50 to 69 years, and 85 patients (34.4%) were 70 years or older. Overall, 50.6% of the study population was male, and 80.6% was white. Median patient age was 63 years. Median follow-up for these patients was 25 months (range 1–319 months). Five-year OS and CSS rates were 47.4% (95% confidence interval (CI), 41.2% to 54.6%) and 58.0% (95% CI, 51.6% to 65.3%), respectively. Actuarial 5-year OS and CSS for all patients are also shown in Table 1.

**Table 1:** **Patient characteristics and 5-year survival**

	Patients		OS		CSS	
Characteristic	No.	%	5 y (%)	95% CI	5 y (%)	95% CI
All patients	247	-	47.4	41.2-54.6	58.0	51.6-65.3
Age						
<50 years	68	27.5	71.3	60.8-83.6	77.3	67.5-88.6
50-69 years	94	38.1	45.7	35.9-58.3	56.4	46.2-68.9
70+ years	85	34.4	29.9	21.1-42.5	42.8	32.3-56.6
Sex						
Male	125	50.6	51.8	43.4-61.9	62.2	53.5-72.2
Female	122	49.4	42.4	33.7-53.4	53.7	44.6-65.6
Race						
White	199	80.6	48.9	42.0-56.9	58.0	51.0-66.1
Non White	48	19.4	40.8	27.9-59.7	57.8	43.9-76.1
Year of diagnosis						
1983-1989	25	10.1	50.7	34.2-75.1	54.0	37.2-78.5
1990-1999	68	27.5	33.4	23.8-46.8	43.7	32.9-58.0
2000-2011	154	62.3	54.0	45.8-63.6	66.2	58.1-75.3
Histologic type						
Squamous cell carcinoma	138	55.9	28.7	21.5-38.4	40.7	32.3-51.3
Adenocarcinoma	34	13.8	73.5	59.1-91.3	83.5	71.1-98.0
Others	75	30.4	68.9	58.6-81.1	76.1	66.1-87.6
Stage						
Localized	68	27.5	74.8	64.4-86.9	84.7	75.8-94.6
Regional	134	54.3	42.0	33.9-52.0	52.1	43.5-62.3
Distant	45	18.2	23.0	13.0-40.9	33.8	20.8-55.0
Surgery						
No	56	22.7	37.7	26.3-54.1	50.9	38.2-67.9
Yes	191	77.3	50.4	43.3-58.6	60.1	53.0-68.3
Radiation						
No	111	44.9	66.7	58.1-76.7	78.5	70.6-87.2
Yes	136	55.1	30.9	23.4-40.8	40.5	32.1-51.1

*Abbreviations*: *OS* Overall survival, *CSS* Cause-specific survival, *CI* Confidence Interval.

Prognosis was worse with increasing age. The 5-year OS was 71.3% for younger patients, 45.7% for the sub-group aged 50 to 69 years, and 29.9% for the oldest group aged 70 years or older. The corresponding 5-year CSS was 77.3%, 56.4% and 42.8% for these three age groups. Over 60% of patients were diagnosed after 2000. Prognosis was better in the most recent decade than in periods before 2000. Five-year OS for patients diagnosed between 2000 and 2011 was 54.0%, and CSS was 66.2%.

Squamous cell carcinoma was present in approximately 55.9% of all patients with the poorest prognosis. The five-year survival rates were 28.7% and 40.7% for OS and CSS, respectively. Other histological subtypes included adenocarcinoma (13.8%), and others (30.4%) with 5-year OS of 73.5%, and 68.9%; and CSS of 83.5% and 76.1%, respectively. Distant disease exhibited the worst prognosis, with 5-year OS being 23.0%, compared with localized and regional disease, with 5-year OS of 74.8% and 42.0%, respectively. The 5-year CSS was 84.7%, 52.1% and 33.8% for localized, regional and distant groups, respectively.

Around 77% patients were treated by surgery. Five-year OS was 50.4% with surgery and 37.7% without surgery, and the corresponding 5-year CSS was 60.1% and 50.9%, respectively. Around 55% patients underwent radiation. The 5-year OS was 30.9% and 66.7% for radiation and no radiation, and 5-year CSS was 40.5% and 78.5%, respectively. Actuarial survival grouped by histological subtype, stage, treatment is shown in Figure 1.

### Univariable and multivariable models

The unadjusted association with prognosis is listed in Table 2. The results of multivariable models are shown in Tables 3 and 4, which show both the full model and the final model. The assumption of a proportional hazard was supported. Results of models predicting OS and CSS showed similar results. In the full model, statistically significant covariates were age, histological subtype, stage, surgery and radiation, according to the Wald test. Histological subtype showed a significant interaction effect with surgery. After model selection, age, stage of tumor, histological type, surgery and radiation treatment were left in the reduced models.

**Table 2:** **Univariate analyses of survival in patients with middle ear cancer**

	Overall survival			Cause-specific survival		
Covariate	HR	95% CI	*P-*value	HR	95% CI	*P-*value
Age (70:45)	2.50	1.86-3.35	<0.001*	2.17	1.53-3.08	<0.001*
Sex						
Male	1			1		
Female	1.13	0.81-1.57	0.46	1.27	0.85-1.90	0.24
Race						
White	1			1		
Non White	1.10	0.73-1.64	0.66	0.86	0.51-1.45	0.56
Year of diagnosis (2000:1990)	0.82	0.65-1.02	0.08	0.72	0.56-0.93	0.01*
Stage						
Localized	1			1		
Regional	2.38	1.52-3.73	<0.001*	3.99	2.04-7.81	<0.001*
Distant	3.80	2.25-6.41	<0.001*	6.46	3.10-13.50	<0.001*
Histological type						
Squamous cell carcinoma	1			1		
Adenocarcinoma	0.24	0.13-0.44	<0.001*	0.20	0.09-0.45	<0.001*
Others	0.34	0.23-0.52	<0.001*	0.31	0.18-0.53	<0.001*
Surgery						
No surgery	1			1		
Surgery	0.59	0.40-0.86	0.01*	0.61	0.39-0.96	0.03*
Radiation						
No	1			1		
Yes	2.51	1.75-3.58	<0.001*	3.55	2.22-5.69	<0.001*

*Abbreviations*: *HR* Hazard Ratio, *CI* Confidence Interval.

*P <0.05.

**Table 3:** **Multivariable analysis of overall survival in patients with middle ear cancer**

	Full model			Reduced model		
Covariate	HR	95% CI	*P-*value	HR	95% CI	*P-*value
Age (70:45)	2.05	1.50-2.80	<0.001*	2.07	1.52-2.82	<0.001*
Sex						
Male	1			-		
Female	1.17	0.82-1.67	0.38	-	-	-
Race						
White	1			-		
Non White	1.11	0.73-1.68	0.64	-	-	-
Year of diagnosis (2000:1990)	0.89	0.70-1.14	0.35	-	-	-
Stage						
Localized	1			1		
Regional	2.01	1.23-3.28	0.005*	2.00	1.22-3.25	0.01*
Distant	3.47	1.94-6.22	<0.001*	3.46	1.93-6.19	<0.001*
Radiation						
No	1			1		
Yes	1.52	1.00-2.30	0.05	1.66	1.12-2.46	0.01*
Histological types^#^	-	-	<0.001*	-	-	<0.001*
Surgery^#^	-	-	0.02*	-	-	0.02*
Histology × surgery	-	-	0.01*	-	-	0.01*
Surgery						
Squamous cell carcinoma	1			1		
Adenocarcinoma	0.23	0.11-0.47	<0.001*	0.24	0.12-0.50	<0.001*
Others	0.67	0.39-1.17	0.15	0.70	0.40-1.20	0.20
No surgery						
Squamous cell carcinoma	1			1		
Adenocarcinoma	1.97	0.55-7.11	0.29	2.21	0.61-7.93	0.22
Others	0.57	0.25-1.27	0.16	0.58	0.26-1.28	0.18

*Abbreviations*: *HR* Hazard Ratio, *CI* Confidence Interval.

^#^Hazard ratios for histology type are presented by surgery because of the significant interaction between these two covariates.

*P <0.05.

**Table 4:** **Multivariable analysis of cause-specific survival in patients with middle ear cancer**

	Full model			Reduced model		
Covariate	HR	95% CI	*P-*value	HR	95% CI	*P-*value
Age (70:45)	1.66	1.16-2.38	0.004*	1.70	1.19-2.45	0.002*
Sex						
Male	1			-		
Female	1.37	0.89-2.11	0.15	-	-	-
Race						
White	1			-		
Non White	0.80	0.47-1.38	0.42	-	-	-
Year of diagnosis (2000:1990)	0.80	0.61-1.05	0.11	-	-	-
Stage						
Localized	1			1		
Regional	2.93	1.43-6.00	0.003*	2.93	1.43-5.99	0.003*
Distant	4.88	2.20-10.82	<0.001*	4.82	2.17-10.69	<0.001*
Radiation						
No	1			1		
Yes	1.84	1.07-3.15	0.03*	2.14	1.28-3.58	0.004*
Histological types^#^	-	-	0.003*	-	-	0.004*
Surgery^#^	-	-	0.06	-	-	0.05
Histology × surgery	-	-	0.03*	-	-	0.02*
Surgery						
Squamous cell carcinoma	1			1		
Adenocarcinoma	0.16	0.06-0.46	<0.001*	0.18	0.06-0.50	0.001*
Others	0.55	0.27-1.12	0.10	0.60	0.30-1.19	0.15
No surgery						
Squamous cell carcinoma	1			1		
Adenocarcinoma	1.95	0.40-9.51	0.41	2.33	0.47-11.68	0.30
Others	0.51	0.19-1.39	0.19	0.54	0.20-1.46	0.22

*Abbreviations*: *HR* Hazard Ratio, *CI* Confidence Interval.

^#^Hazard ratios for histology type are presented by surgery because of the significant interaction between these two covariates.

*P <0.05.

### Development of prognostic nomogram

**Figure 2:**
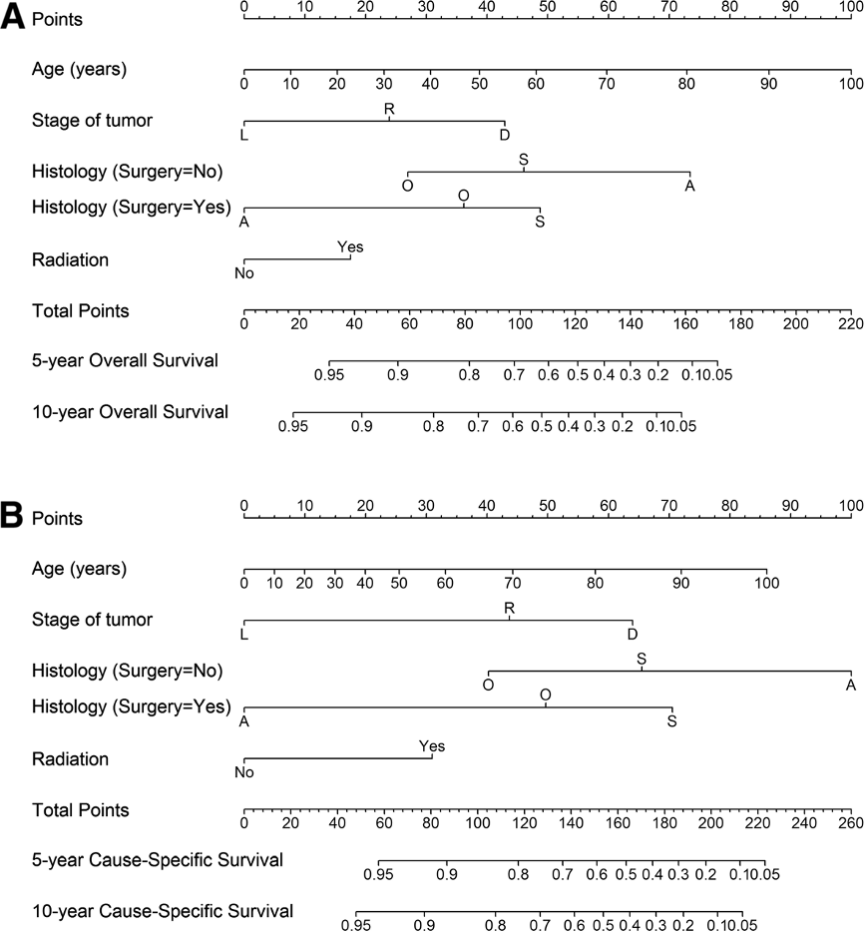
**Nomograms for predicting 5- and 10-year overall survival and cause-specific survival. (A)** Prediction for overall survival; **(B)** Prediction for cause-specific survival. Abbreviations: Stage: L, localized; R, regional; D, distant. Histological subtype: S, squamous cell carcinoma; A, adenocarcinoma; O, others. Instructions: Locate the patient’s characteristic on the variable row and draw a vertical line straight up to the points row to assign a value of points for the variable. Repeat this process to obtain points for each variable. Add up the total points and drop a vertical line from the total points row to obtain the 5- and 10-year survival.

The nomograms were developed for predicting OS and CSS based on beta coefficients in finial models (Figure 2). To use the nomogram, first draw a vertical line up to the points row to assign points for each variable, then add up the points for each variable to obtain the total points, and drop a vertical line from the total points row to obtain the 5- and 10-year survival.

**Figure 3:**
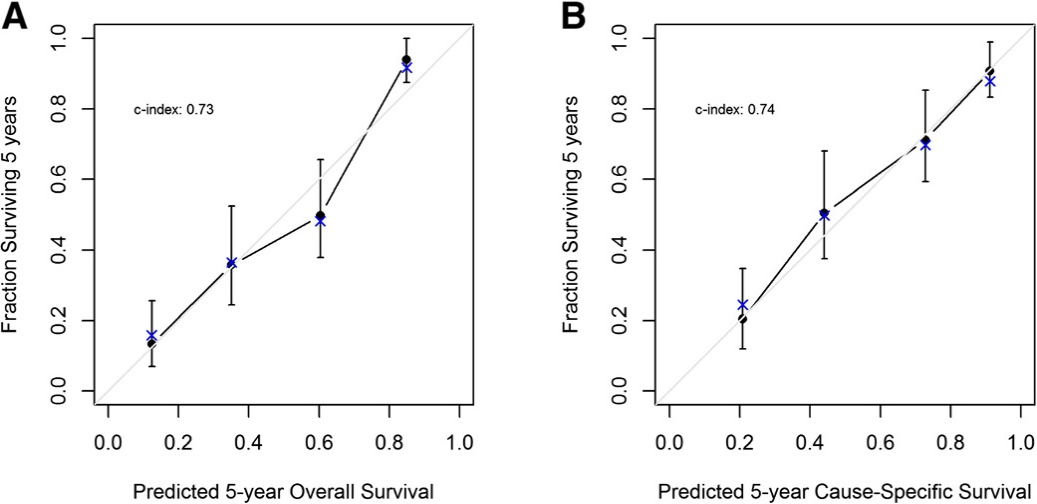
**Calibration plots. (A)** Overall survival; **(B)** Cause-specific survival. The grey line represents the “ideal” line of a perfect match between predicted and observed survival. Dots correspond to apparent predictive accuracy. X marks the bootstrap corrected estimates.

Model performance was evaluated by internal validation with bootstrapping. The discrimination (bootstrap-corrected concordance index) was 0.73 for model predicting OS, and 0.74 for CSS. This implies that the models are reasonably accurate. The calibration plots are shown in Figure 3. Points in both plots close to the 45-degree line show good agreement between predicted and observed outcomes.

## Discussion

We estimated the survival of primary middle ear cancer and developed prognostic models to predict 5-year OS and CSS using a SEER dataset. Simple nomograms were constructed based on the prognostic model, which included five variables available from the cancer registry or routine clinical practice.

Individual estimation of survival probability for cancer patients is useful for treatment selection and clinical counseling. An individual predictive value of prognosis can also be used to identify and stratify patients for clinical trials. A nomogram based on a statistical model provides clinicians and patients with a practical tool for prognostic prediction [9]. A number of important cancer prognostic models and nomograms have been developed and are in use today for prostate, pancreas, breast, and thyroid cancer and other cancer sites [10–15]. To our knowledge, the present nomogram is the first to use the SEER database to predict the prognosis of middle ear cancer.

Because of the rarity of middle ear cancer, evaluation of prognosis can be challenging. Most of the literature has reported results combining malignancies of external auditory canal with primary tumors of the middle ear. So far, middle ear cancer as a subgroup of malignant tumor of the temporal bone has not been well studied. In addition, most published studies involving the temporal bone originate from the experiences of single institutions and the results are heterogeneous. Due to the short follow-up periods and rare cases and events, reports from a single institution often do not have sufficient power to identify true prognostic factors. A population-based study can provide more reliable analysis, and the results are likely to be more generally applicable. The SEER data are a powerful tool for exploring prognostic factors, especially for unusual and rare tumors.

The simplicity of our prognostic model is also a strength. In clinical practice, complex models may not be well accepted and implemented. Our nomogram is based on few predictors, which are all available from routine clinical work. We therefore believe that it can be easily used by clinicians to make accurate individualized prognosis estimates.

Richard first reported the prognosis of middle ear cancer using the SEER database [2], showing 5-year OS by stage, histology and treatment of patients diagnosed between 1973 and 2004. However, only univariate analysis was conducted in Richard’s study. In this article, we updated their results to 2011 and added multivariable models and nomograms to predict OS and CSS.

Stage was found to be an independent prognosis factor of overall survival, with local patients having better survival than regional and distant patients. We did not use a staging system currently used for temporal bone malignancy, such as the Pittsburgh staging system, which provides a comprehensive means of assessing temporal bone tumors according to imaging and preoperative clinical information. The Pittsburgh staging system is based on external auditory canal malignancies, [1] and therefore was not suitable for staging cancer in this study. Stell and McCormick proposed a staging system in 1985 that can be used for staging both external auditory canal and middle ear cancer [16]. Nevertheless, the Stell and McCormick staging system also could not be used in this study because of the limited SEER dataset.

Surgical resection with the purpose of achieving a negative margin and decreasing morbidity or mortality is considered to be the standard of care for middle ear cancer. It is agreed that all patients who are able to tolerate an operation should be treated with surgery, except those who are diagnosed with histiocytosis X, which is treated primarily with radiation only or adjuvant chemotherapy [1]. Surgical approaches used in clinical practice include local canal resection, sleeve resection, en bloc resection of the external auditory canal, lateral temporal bone resection, subtotal temporal bone resection and total temporal bone resection [17]. Whether radical surgery is necessary remains under debate [1]. Unfortunately, SEER does not provide data regarding the detailed surgical approach and margin status. Further evaluation of the surgical management of middle ear cancer needs other cohorts or series.

Radiotherapy is advocated as an adjunct to surgery or for palliation, rather than as a curative approach. T2 and higher-staged tumors, recurrent tumors, positive margins, perineural spread, positive lymph nodes, or extracapsular spread are indications for postoperative radiotherapy. The effectiveness of adjuvant radiation remains controversial. Some studies have demonstrated an improvement in terms of the survival rate and local control in patients with positive surgical margins who underwent adjuvant radiotherapy compared with patients who underwent surgery only [18–20]. In contrast, other authors concluded that a positive surgical margin was the major cause of recurrence, and adjuvant radiotherapy showed no more effect on survival [21–23]. Results of our study suggested that radiotherapy may be an adverse prognostic factor for survival. Patients who received radiotherapy likely have inoperable disease, co-morbid disease, or a positive surgical margin; thus, their prognosis was worse. Because this study was limited to the predictive factors available from the SEER database, these factors could not be adjusted in the current model. New sources of data which consider a more detailed extent of disease or stage will be important to address this issue. Furthermore, due to the lack of data on local recurrence, the survival benefit of adjuvant radiotherapy for loco-regional control may have been missed.

There are several limitations of this study. First, some factors impacting survival are not included in our models. For example, it appears that positive margins and promontory or facial nerve involvement are negative prognostic markers. However, this surgical information cannot be found in the SEER registry. In addition, chemotherapy data cannot be obtained from the public SEER database. Second, patients were enrolled in the database over three decades. It is therefore possible that improvements in multiplanar imaging, Intensity Modulated Radiation Therapy (IMRT), and chemotherapy, along with advances in skull base surgical techniques have combined to improve overall survival. We did not use the full model to build the nomogram, which was adjusted for the year of diagnosis. After model selection, the year of diagnosis showed drop outs. Thus, our final models and nomograms might have a low estimate of survival probability. Although this low estimate of prognosis might exist, it was not serious because over 60% of patients in the study cohort enrolled after 2000. Finally, we used internal validation in the model due to the relatively small sample size. Examination of the apparent accuracy of a multivariable model using the training dataset results in optimistically biased performance [24], and the potential for optimism in model performance increases when the number of events decreases and the number of candidate predictors increases [25]. At here, we used the bootstrapping technique to obtain a nearly unbiased internal assessment of accuracy. However, it is not enough to demonstrate a good performance of a model on the development dataset only, even after corrections from bootstrapping [26], external validation is still need to confirm whether our nomograms can be generalized to a new patient population. Despite its limitations, SEER still provides the largest cohort of middle ear cancer patients available, which is a valuable resource for providing valid statistical comparison and building predictive models for this rare lesion.

## Conclusions

In conclusion, we analyzed prognostic data on middle cancer using the SEER database, a high quality reliable cancer cohort. Survival outcome specific to histology, stage, surgery, radiation therapy and other prognostic factors are described. We then built survival models to predict 5- and 10-year OS and CSS. The models provide objective analysis of all currently available data. The performance of the models is good, with a c-index of 0.73 and 0.74 for models predicting OS and CSS. These nomograms should thus be considered as an accurate tool to predict the clinical prognosis.

## Abbreviations

LHX: Langerhans histocytosis X
SEER: Surveillance epidemiology and end results
AJCC: American joint committee on cancer
Rms: Regression modeling strategies
OS: Overall survival
CSS: Cause-specific survival
AIC: Akaike information criterion
c-index: concordance index
CI: Confidence interval
IMRT: Intensify-modulated radiation therapy
L: Localized
R: Regional
D: Distant
S: Squamous cell carcinoma
A: Adenocarcinoma
O: Others.
